# Recent Advances in Target Characterization and Identification by Photoaffinity Probes

**DOI:** 10.3390/molecules180910425

**Published:** 2013-08-29

**Authors:** Jitapa Sumranjit, Sang J. Chung

**Affiliations:** 1 BioNanotechnology Research Center, KRIBB, 125 Kwahangno, Yuseong, Daejeon 305-806, Korea; 2 National Nanotechnology Center (NANOTEC), 111 Phahonyothin Rd., Klongluang, Pathumthani 12120, Thailand; 3 Department of Chemistry, Dongguk University, 26 Phil-dong, Jung-gu, Seoul 100-715, Korea

**Keywords:** protein target identification, photoaffinity labeling, affinity chromatography, chemical proteomics, photo-crosslinking, bioorthogonal ligation, two step labeling, click chemistry

## Abstract

Target identification of biologically active molecules such as natural products, synthetic small molecules, peptides, and oligonucleotides mainly relies on affinity chromatography, activity-based probes, or photoaffinity labeling (PAL). Amongst them, activity-based probes and PAL have offered great advantages in target identification technology due to their ability to form covalent bonds with the corresponding targets. Activity-based probe technology mainly relies on the chemical reactivity of the target proteins, thereby limiting the majority of the biological targets to enzymes or proteins which display reactive residues at the probe-binding site. In general, the probes should bear a reactive moiety such as an epoxide, a Michael acceptor, or a reactive alkyl halide in their structures. On the other hand, photoaffinity probes (PAPs) are composed of a target-specific ligand and a photoactivatable functional group. When bound to the corresponding target proteins and activated with wavelength-specific light, PAPs generate highly reactive chemical species that covalently cross-link proximal amino acid residues. This process is better known as PAL and is widely employed to identify cellular targets of biologically active molecules. This review highlights recent advances in target identification by PAL, with a focus on the structure and chemistry of the photoaffinity probes developed in the recent decade, coupled to the target proteins identified using these probes.

## 1. Introduction

**Scheme 1:**
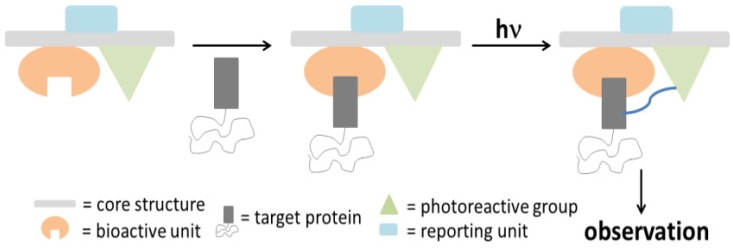
Cartoon representing the general overview of a photoaffinity labeling process.

Photoreactive molecules are being continuously developed for a variety of applications. Amongst them, photoaffinity compounds were first introduced by Westheimer in the early 1960, as a concept of photoaffinity labeling (PAL) [1]. This concept is proven to be an efficient approach to identify various target proteins rendering the design, synthesis, and application of photoaffinity compounds beneficial in the fields of medicinal chemistry and chemical biology [2] A typical photoaffinity probe (PAP) contains three functionalities, namely a pharmacophore (a target-specific ligand), a photo reactive group and a reporter tag [3]. During the PAL process, a ligand is covalently attached to a photoreactive group, which generates a reactive species upon irradiation that covalently cross-links the ligand to its target macromolecule (Scheme 1). Detailed examples of photoaffinity utilization include interrogation of the structure and function of biological molecules, identification of the targets of biological active compounds, determination of the selectivity and affinity of ligand-target complexes, isolation and identification of unknown enzymes or receptors, investigation of ligand-receptor interactions, identification of amino acid residues at protein-protein, and proteins-lipid interfaces [4]. 

The recent advances in the design and synthesis of PAPs will be covered in this review. The common photoreactive compounds, based on benzophenones, arylazides and diazirines, which have found widespread application as photoreactive groups in the PAL process, will be included. In addition, the identification of the biological targets by the PAL probes will be discussed.

## 2. Photoaffinity Probes (PAPs)

**Figure 1:**
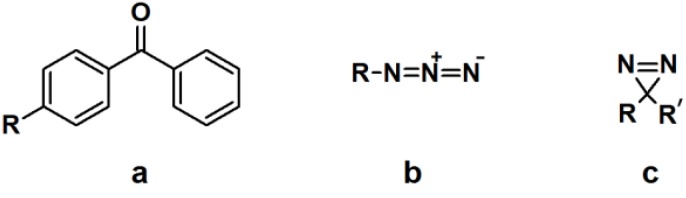
Commonly used photoreactive units: (**a**) benzophenone; (**b**) aryl azide and (**c**) diazirine.

Ideally, a PAP should meet the following criteria: (1) possess high stability in the dark under various pH conditions; (2) bear a structural resemblance to the target molecule, with similar affinity; (3) be sterically non-congested; (4) have a wavelength-selective activation which does not result in damage to other components in the system; (5) generate highly reactive (short-lived) photo-intermediate(s) upon irradiation; (6) have the ability to react with any types of bond or residue without any preference; and (7) form a stable adduct with the target receptor in order to survive detection methodology [5,6]. However, thus far there have been no reports of probes possessing all the aforementioned characteristics. The typical components of a PAP are a photoreactive group, a target binding ligand, and a reporter tag. The most important component which will be discussed and used to categorize the photoaffinity probes is the photoreactive moiety. Three major types of photoreactive groups are commonly used in PAL, namely benzophenones, arylazides, and diazirines (Figure 1). 

**Scheme 2:**
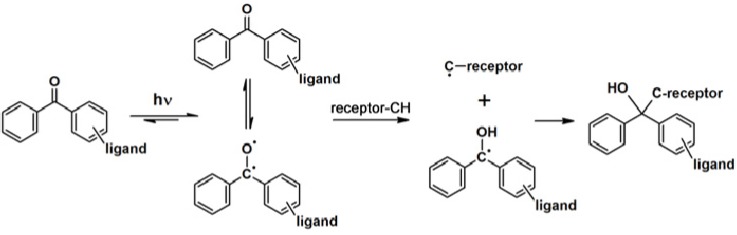
Photoactivation mechanism of benzophenone [6].

Benzophenone-based PAPs are usually activated with light at wavelengths around 350–360 nm, which limits protein degradation and enables studies on cell cultures or other living systems. A number of building blocks based on benzophenone are commercially available. Upon irradiation, benzophenone generates reactive triplet carbonyl states (Scheme 2) which can react with inactive C–H bonds [6]. The advantages of benzophenones include their stability in most organic solvents and their compatibility with several synthetic strategies. However, the major drawbacks of benzophenone- derived probes are that their bulkiness could hinder the binding with their targets and their normal requirement for long irradiation times, during which non-specific labeling can occur [1,7,8]. 

**Scheme 3:**
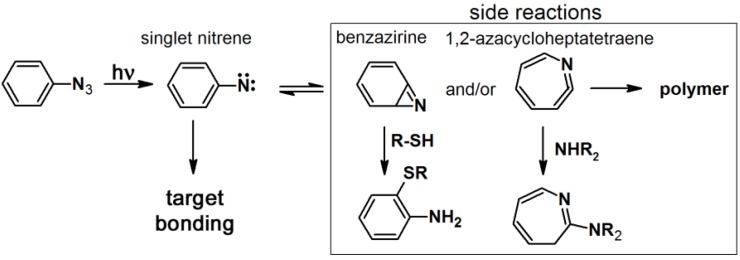
Photoactivation mechanism of aryl azide [6].

Aryl azides are most frequently used as PAPs because they are small molecules which often are readily synthesized. Furthermore, aryl azides are relatively stable in the dark and highly reactive upon photoirradiation. However, the maximum absorption wavelength is less than 300 nm, which can induce severe damage to biological systems upon irradiation. The activation of an aryl azide generates the desired singlet nitrene (Scheme 3) which can generate a triplet nitrene via intersystem crossing. Moreover, at a certain temperature, the singlet nitrene rearranges into a bicyclic benzazirine, which generates 1,2-azacycloheptatetraene that can interact with remote nucleophiles thereby decreasing the photolabeling yields and inducing non-specific labeling [1,9,10]. 

**Scheme 4:**
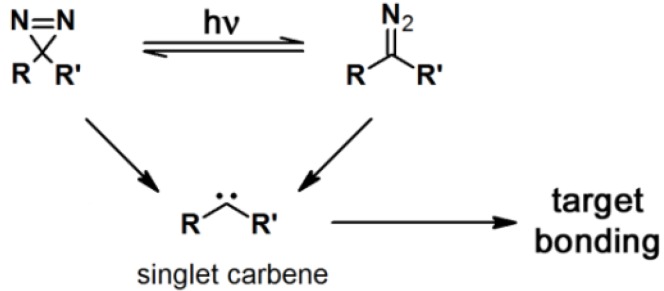
Photoactivation mechanism of diazirine derivatives [5].

Diazirine derivatives are the smallest molecular size PAPs amongst the common photoreactive units. These derivatives are quite stable at room temperature, highly reactive upon photoactivation and relatively inert towards nucleophilic attack, acidic- and alkaline conditions. Moreover, the excitation wavelength for diazirine-derived PAPs is in the range of 350–380 nm, which is not harmful for most biopolymers. Upon irradiation, diazirine generates a reactive carbene (Scheme 4) which rapidly forms a covalent bond with the nearest target molecule via C–C, C–H, O–H, or X–H insertion. The generated carbenes are easily quenched by water, resulting in diminished labeling yields. However, the specificity of the labeling is high because only the desired target protein can reach a proximity which is enough to react with the specific probe during the short life-time of the carbene. One of the drawbacks of diazirine derivatives is that their synthesis requires long and complicated synthetic procedures. A point of caution in designing PAPs is that a small modification can significantly alter the essential features of the ligand, which in turn affects its biological activity [1,5,11,12].

In order to evaluate and identify protein labeling, certain reporting moieties are incorporated into the PAP. Typical reporter tags include radioactive isotopes, biotin, epitope tags, or fluorophores. These reporting units are usually covalently attached to the probes; however, their bulkiness and/or instability could perturb the protein binding process. Therefore, several bioorthogonal processes were developed as an alternative [13,14]. In one example of this new molecular design, the reporting group is replaced by either a terminal alkyne or an aliphatic azide, which can be readily subjected to a copper-catalyzed (or copper free) Hüisgen 1,3-dipolar cycloaddition, or ‘click’ reaction [15,16]. The reactants are functionalized with a reporter tag such as biotin or a fluorophore and decorated with the complementary alkyne or azide [17,18]. After cross-linking of the probe with the target protein upon photoirradiation, the complex is subjected to a ‘click’ reaction with the corresponding reporting group and subsequently analyzed with the appropriate protocol. Much effort has made recently into designing the aforementioned bifunctional probes to facilitate target enrichment. The challenge to the successes in this approach, however, lies with minimizing non-specific incorporation of the probes into non-target proteins. The evaluation of the labeling process can be achieved by a variety of different methods, depending on the type of the employed reporter tag. For example, fluorophores can be detected by in-gel fluorescence emission, while the conjugated biotin probe is subjected to western blot analysis.

### 2.1. Benzophenone-Based Photoaffinity Labeling

The synthesis of a benzophenone-based PAP can readily be achieved by linking commercially available functionalized benzophenone derivatives to the probe. Otherwise, the benzophenone motif can be synthesized from a suitable precursor bearing the desired linking unit. The utilization of benzophenone-derived affinity probes has been widely explored, revealing both its advantages and disadvantages. This article will deal with some recent published studies.

γ-Secretase is an integral membrane protease that cleaves the amyloid precursor proteins (APP) to release Aβ peptides, which have a causative role in the pathogenesis of Alzheimer’s disease (AD) [19,20]. γ-Secretase is a complex of four different integral membrane proteins (presenilin, nicastrin, Aph-1 and Pen-2). The heterogeneity within the subunit composition has hampered the isolation of purified enzyme for subsequent structural biology studies. Therefore, there is a high demand for the development of methods to target γ-secretase activity in order to lower the levels of Aβ42 production without blocking the overall processing of γ-secretase substrates. 

**Figure 2:**
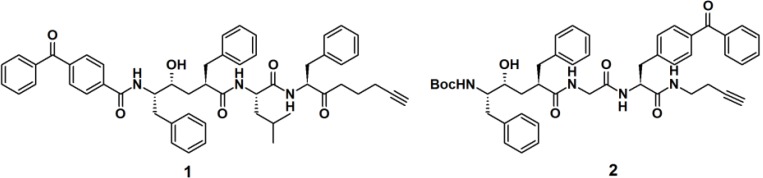
Benzophenone-based PAPs **1** and **2** for γ-secretase recognition.

Crump *et al.* described the synthesis of terminal alkyne-functionalized benzophenone-based affinity probes **1** and **2** to target presenilin–1 N-terminal fragment (PS1-NTF) (Figure 2) [19]. Both probes are potent γ-secretase inhibitors. The probes were synthesized by the coupling of benzophenone-4-carboxylic acid with a ligand-binding unit via amide bond formation. Instead of the reporting group, a terminal alkyne was incorporated as a ligation handle to enable click reactions with various kinds of azide-functionalized reporter tags, after the probe is cross-linked to the target. The PAL potencies of the probes were determined by the incubation with HeLa cell membranes and subsequent irradiation with light with a wavelength of 350 nm. Next, the labeled proteins were subjected to biotin-azide or TAMRA-azide (a rhodamine-based dye) ligation and subsequently visualized via the appropriate method, western blot analysis and in-gel fluorescence detection, respectively. Both probes demonstrated efficient labeling of the target proteins.

**Figure 3:**
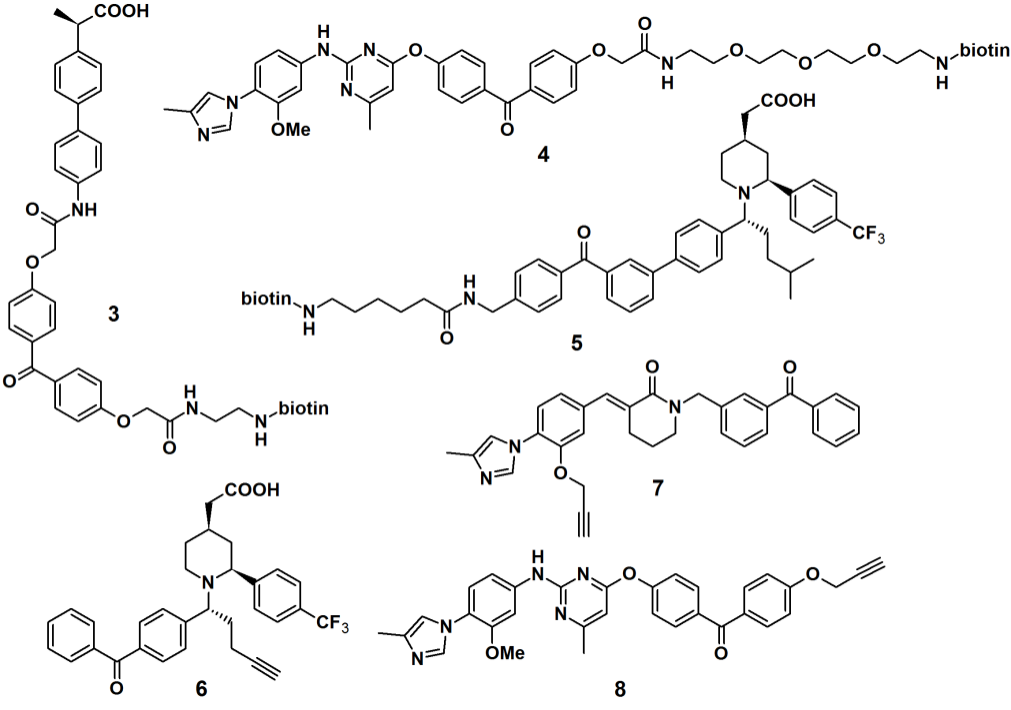
γ-Secretase modulators photoaffinity probes **3**–**8**.

Another approach to target γ-secretase by the use of PAPs has been reported (Figure 3). γ-Secretase modulators (GSMs), originally derived from nonsteroidal anti-inflammatory drugs (NSAIDs), were utilized for this purpose. Carboxylic acid- and heterocyclic GSMs specifically target presenilin, the catalytic subunit of γ-secretase. In addition, these two types of GSMs have distinct binding sites within the γ-secretase complex and exhibit different Aβ profiles. Due to these beneficial properties, GSM-based PAPs were synthesized to determine their efficacy as PAPs. The first generation of these probes (PAPs **3**, **4** and **5**) usually employed biotin as a reporting unit [19,21]. However, the bulky biotin group could reduce the potency of the parental compounds; hence the smaller alkyne functionality is used as a latent reporting group, which facilitates the conjugation of a biotin or fluorescent tag by the ‘click’ reaction in a later stage. The newly developed probes comprised of a GSM pharmacophore, a photoactive benzophenone derivative and an alkyne functionality, similar as described for probes **6**, **7** and **8**. PAP **6** was incubated with PS1ΔE9 proteoliposomes at 37 °C for 1 h, followed by light-mediated activation at 350 nm [22]. The resulting cross-linked proteins were conjugated to tetramethylrhodamine dye by the use of the copper-catalyzed azide-alkyne cycloaddition. Results obtained from sodium dodecyl sulfate-polyacrylamide gel electrophoresis (SDS-PAGE) separation and fluorescent emission readout indicated that the probe specifically and directly interacted with PS1ΔE9 in the reconstituted proteoliposome system.

**Figure 4:**
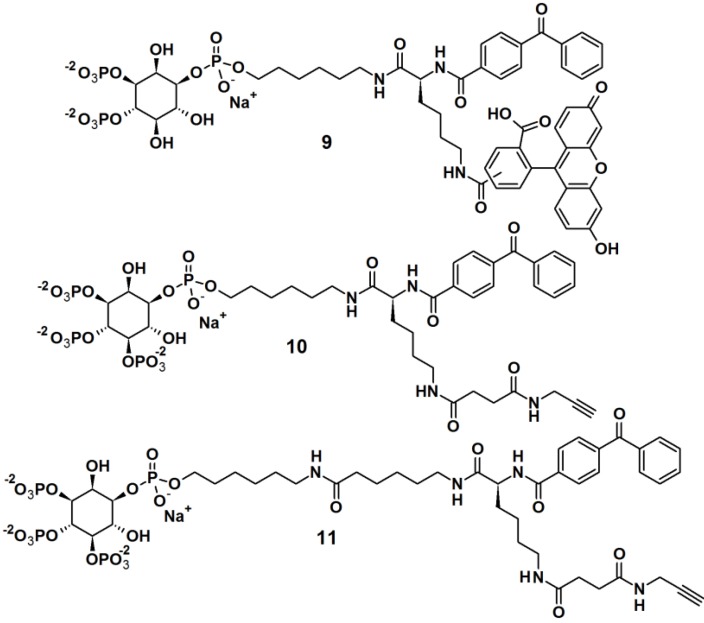
Benzophenone-based photoaffinity probes **9**–**11**.

Rowland *et al.* [23] reported the synthesis of bifunctional PAPs **9**, **10** and **11**, *i.e.*, each PAP contains a photoaffinity tag for cross-linking to proteins and a secondary tag for subsequent analysis (Figure 4). These probes employed phosphatidylinositol polyphosphates (PIP_n_s), which are lipids that regulate critical biological processes related to diseases, and also act as site-specific ligands in interactions that enforce membrane association of protein binding partners. In this report, PI(3,4,5)P_3_ was incorporated as a ligand to target cancer cells. While the PIPs head group is hydrophilic in nature, the hydrophobic primary alkane moiety imitates the lipophilicity of the glycerolipid backbone. This strategic design ensured that the affinity probe will not be embedded and overshadowed by the membrane core. The probes were successfully tested in labeling studies with a purified protein, the PH domain of Akt, which could be observed by in gel-detection. In the reported study, probe **10**, with a shorter linker, resulted in a higher protein labeling compared to those having a shorter linker. This observation led to the utilization of probe **10** in proteomic labeling studies using cell extracts. The labeled proteins were visualized by in gel-detection and characterized using post-labeling experiment with biotin, affinity chromatography, and tandem mass spectrometry. The studies yielded a total of 265 proteins, including both known and novel PI(3,4,5)P_3_-binding proteins candidates.

**Figure 5:**
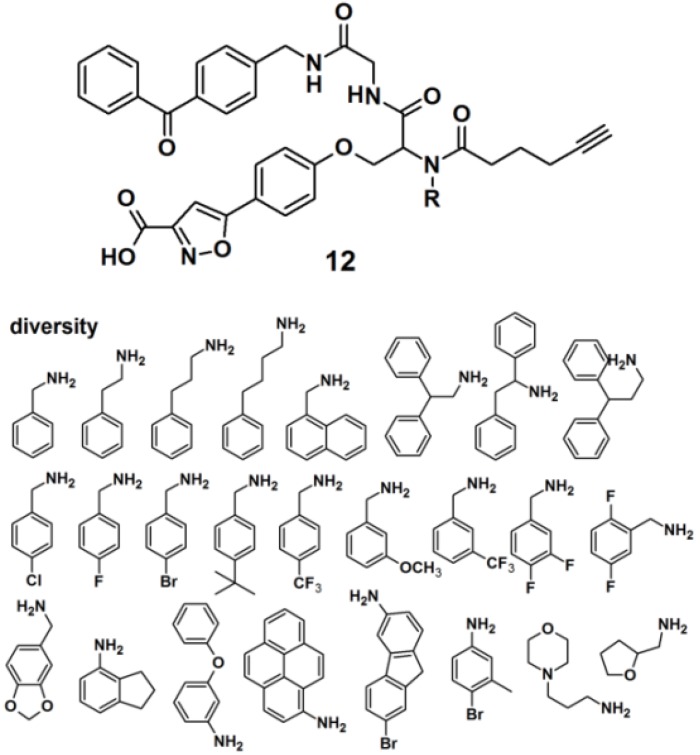
Benzophenone-based photoaffinity probes **12** for PTPs labeling.

Protein tyrosine phosphatases (PTPs) are also immensely studied target proteins. Twenty five PAPs with the general structure as depicted for probe **12**, were efficiently synthesized to target PTPs by the use of a multiple-component reaction (MCR) (Figure 5), demonstrating that the MCR approach is successful and time-saving in the development of novel PAPs [24]. The designed probes were constructed out of four parts, namely a photoreactive unit, a ‘click’ handle and two binding sites for a bidentate target, since it has recently been discovered that many PTPs possess a highly distinct secondary binding site, in addition to the primary active site where phosphotyrosine (*p*Tyr) binds [25]. The applied MCR, the Ugi reaction, employs the following components: (1) an isoxazole carboxylic acid decorated with an aldehyde, which mimics *p*Tyr and binds to the PTP primary active site; (2) an amine-component which potentially targets the secondary binding site on PTPs; (3) an isonitrile-functionalized benzophenone motif to initiate cross-linking of PTP and the probe upon UV irradiation, and (4) a terminal alkyne-modified carboxylic acid to serve as a ‘click’ handle to facilitate subsequent in-gel fluorescence scanning and pull-down experiments by conjugation to suitable azide-decorated reporter. It is noteworthy to report that reporter tags such as dye-N_3_ and biotin-N_3_ were not directly attached to the probe because their bulkiness could interfere with the protein binding. The obtained probes were successfully used to label endogenous PTP1B in mammalian cell lysates.

**Figure 6:**
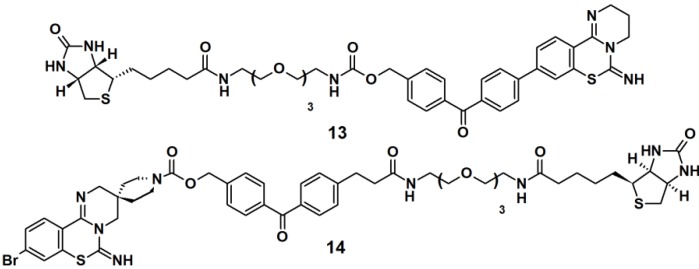
Benzophenone-based photoaffinity probes **13** and **14**.

Benzophenone-based PAPs **13** and **14** (Figure 6) were designed to study the target for 3,4-dihydro-*2H*,*6H*-pyrimido[1,2-c][1,3]benzothiazin-6-imine (PD 404182), a potent antiviral agent against the human immunodeficiency virus (HIV) [3]. The designs of these probes were based on the following characteristics: (1) the introduction of a hydrophobic- and photoreactive benzophenone analog onto the pyrimidobenzothiazine scaffold; (2) the incorporation of an *N*-alkoxycarbonyl piperidine ligand onto the amidine substructure in order to achieve potent anti-HIV activity. Additionally, the latter part can serve as an attachment point for a row of functional groups. The synthesis commenced with the construction of the benzophenone moiety from a derivatized benzoic acid. Next, the antiviral ligand, pyrimidobenzothiazine, was coupled to the benzophenone core, and finally a biotin-functionalized polyethyleneglycol (PEG) linkage was incorporated. Next, the newly synthesized probes were evaluated as photoaffinity labels for HIV-1-infected H9 cells (H9IIIB). Each probe was incubated with the H9IIIB cells before exposure to UV-Vis light. Subsequently the cell lysate was subjected to separation by SDS-PAGE followed by western blot analysis. The obtained results revealed that only probe **13** is useful for the identification of the target protein, PD 404182.

**Figure 7:**
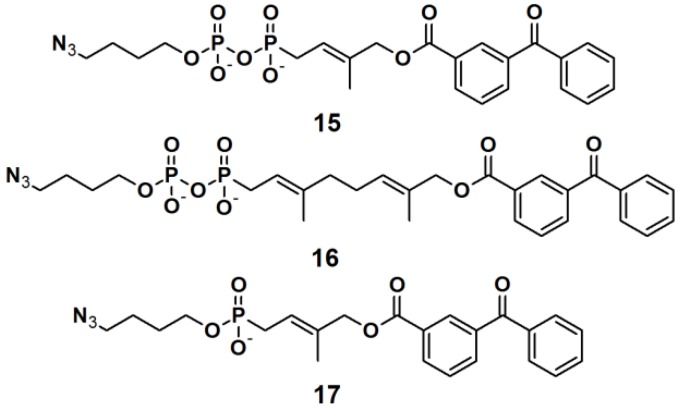
Benzophenone-based photoaffinity probes **15**–**17**.

Three PAPs containing an isoprenoid motif and an azide functionality were recently reported (Figure 7) [26]. Since the isoprenoid chain is enzymatically transferred to proteins via a process known as protein prenylation, this interaction has been utilized as a basis for protein recognition. PAPs **15**, **16** and **17** were designed to bear both, a photoreactive benzophenone- and an azide group, tethered via a short alkyl chain, as attachment side for a reporter tag. To demonstrate the photoaffinity capability of these probes, freshly prepared *Saccharomyces cerevisiae* proteome was incubated with the three probes followed by near UV light-mediated activation at 365 nm. Subsequently, a biotin-functionalized alkyne was used in the click chemistry ligation. The samples were subjected to SDS-PAGE analysis followed by visualization using Coomassie Blue staining and streptavidin blotting. The PAL was successfully demonstrated for all three probes, with similar results, indicating the common interaction of the protein and the isoprenoid motif.

**Figure 8:**
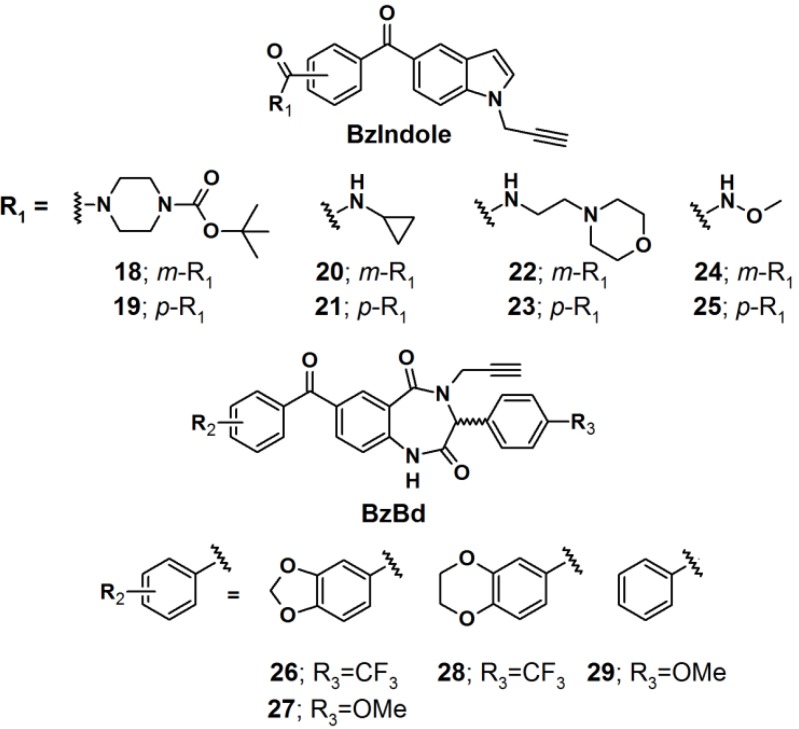
Benzophenone-based photoaffinity probes **18**–**29**.

Two benzophenone-based scaffolds, 5-benzoyl indole (BzIndole) and 7-benzoyl-benzo-1,4-diazepin-2,5-dione (BzBd), were designed as photoreactive core units (Figure 8) [27]. Structural diversity was introduced at multiple sites on these scaffolds in order to generate a library of PAPs intended for protein identification. In this library, each compound contained a terminal alkyne spacer as a click chemistry handle to enable post visualization, enrichment, and identification of the interacting proteins. Each probe was incubated with the human breast cancer cell line MDA-MB-231, photocrosslinked *in situ* with near UV light with a wavelength of 365 nm. The cells were subsequently lysed and their proteomes were conjugated to rhodamine-azide16, under click-chemistry conditions, followed by visualization using SDS-PAGE electrophoreses and fluorescent emission analysis, where the individual probes displayed obviously and distinct protein labeling events. Interestingly, these observed *in situ* profiles differed considerably in comparison to small molecule-proteome reactions, performed *in vitro*, indicating that the probes interact with different sets of proteins in living cells *versus* cell lysates.

Next, the probes were screened for antiproliferative activity in MDA-MB-231 cells under both normal and low glucose conditions. The former condition resembled standard cell culture protocols, while the latter could be a closer mimic of the nutrient-deprived state encountered in solid tumors. While none of the compounds affected MDA-MB-231 proliferation at normal glucose levels, probe **18** greatly inhibited proliferation at low glucose concentrations. The antiproliferative activity of probe **18** was also observed in several other human cancer cell lines.

**Figure 9:**
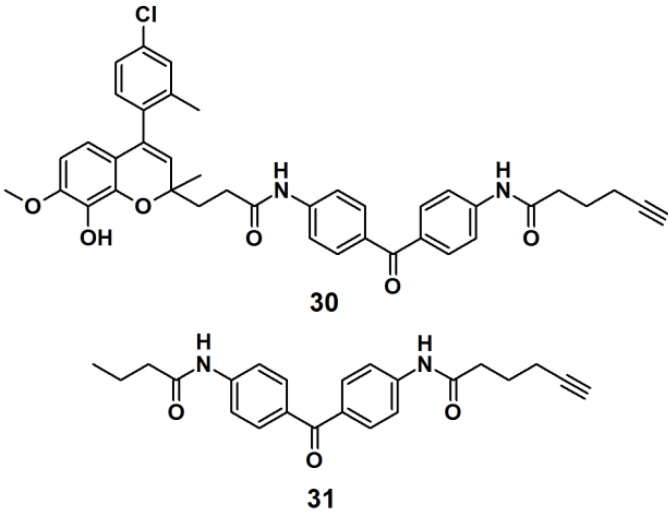
Benzophenone-based photoaffinity probes **30**, **31**.

Target identification in cell lysates tends to differ compared to live cells, mainly due to the non-specific interactions caused by matrices in live cells. To overcome this limitation, a new method called fluorescence difference in two-dimensional gel electrophoresis (FITGE) has been developed and employed in the target identification of a new antitumor agent [28]. PAP **30** (Figure 9) was designed to contain an antiproliferative moiety toward HeLa (human cervical cancer cell line) cells with an IC_50_ of 450 nM, as well as U266- (human myeloma cell line), A549- (human lung cancer cell line), and MCF7 (human breast cancer cell line) cells. The desired probe was constructed of a photoreactive benzophenone derivative and a terminal acetylene group, to enable ‘click’ reaction-based ligation. Probe **31** was prepared and used as a negative control, in order to evaluate and correct for non-specific protein labeling. Both probes were separately incubated with both, cell lysates and live cells. After irradiated with near UV light with a wavelength of 365 nm, probes **30** and **31** were conjugated to the fluorescent dyes, Cy5 and Cy3, respectively, by the use of the click reaction. The labeled proteomes were subsequently subjected to FITGE analysis. Merging of the image obtained from the Cy5 and Cy3 channels revealed three fluorescent colors, red and green spots from proteins preferentially labeled with probe **30** and negative probe **31**, respectively, and yellow spots originating from dual-labeled proteins with probes **30** and **31**. With the efficient exclusion of nonspecific protein labeling using FITGE, the experiments in live cells revealed that probe **30** could be a potent antitumor agent which functions by the inhibition of tubulin polymerization.

### 2.2. Arylazide-Based Photoaffinity Labeling

The synthesis of arylazide-containing PAPs is usually accomplished by subsequent attachment of each functional unit to an arylazide scaffold [10]. However, the terminal aromatic azide can be generated from an aromatic amine under conventional diazotization conditions, NaNO_2_/AcOH-H_2_O/NaN_3_, or *t*-BuONO and TMS-N_3_ in CH_3_CN [29]. 

**Figure 10:**
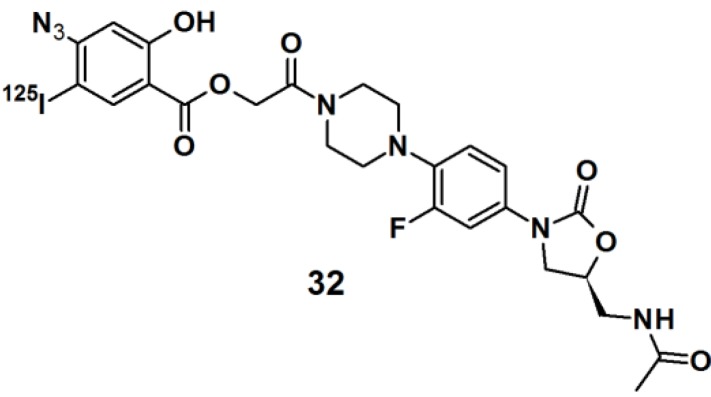
Arylazide-based photoaffinity probes **32**.

Oxazolidinones belong to a class of synthetic antibiotics that inhibits protein synthesis by interfering with ribosomal function [30]. In order to determine the site of oxidazolinone interaction in living cells, ^125^I-labeled arylazide-based PAP **32** was prepared (Figure 10). *Staphylococcus aureus* cells were incubated with this probe and irradiated with UV light with a wavelength of 254 nm. The conjugated cells were lysed and the RNA was extracted. The RNA was then subjected to primer extension and analyzed by two-dimensional electrophoresis analysis. The precise location of the cross-link was determined from RNA mapping which revealed that a binding site for the oxazolidinone antibiotics was located at or near the peptidyltransferase center in actively bacterial ribosomes translation.

**Figure 11:**
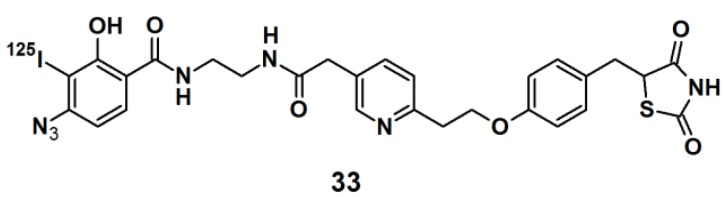
Arylazide-based photoaffinity probes **33**.

Thiazolidinedione (TZD) insulin sensitizers are proven to be effective therapeutic agents for treating a root cause of diabetes. The analog itself is photoreactive and can cross-link to the target protein upon photoirradiation [31,32]. Hence, PAL along with mass spectrometry-based proteomics were applied to identify a previously uncharacterized mitochondrial complex that specifically recognizes TZDs. Probe **33** (Figure 11) was synthesized and labeled with ^125^I. The probe was incubated with mouse liver mitochondrial membranes, followed by photo-irradiation and subsequently analyzed with SDS-PAGE and mass spectrometry. The results indicated that the insulin sensitizing TZDs have a recognition site in the inner mitochondrial membrane which is constructed out of a protein complex that is involved in mitochondrial pyruvate import. The complex was named mTOT (mitochondrial target of thiazolidinones). The key members of mTOT, Mpc1 (BRP44 Like) and Mpc2 (BRP44), are highly conserved proteins, which facilitates pyruvate transport into the mitochondrial matrix.

**Figure 12:**
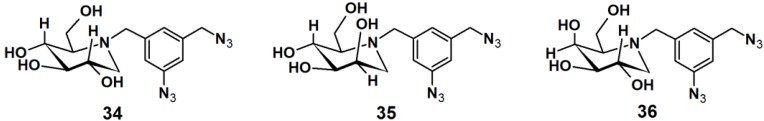
Arylazide-based photoaffinity probes **34**–**36** for exo-α-glycosidases.

Gandy *et al.* reported the development of PAPs to detect exo-α-glycosidases. These glycoside hydrolases are enzymes that degrade the carbohydrate portions of glycoconjugates (Figure 12) [33]. This class of enzymes is found throughout nature, in organisms ranging from bacteria to humans, and involved in a range of biological processes. Therefore, the rapid detection of these proteins is of utmost interest. Affinity-based methods for profiling the proteome for protease and esterase activities is well studied, however only a limited number of report deals with the profiling of specific glycosidase activity. The reported strategy for the design of PAPs utilizes iminosugar-based pharmacophores, which are known to be potent, broad band inhibitors of this class of enzymes. An aromatic azide moiety was chosen as the photoreactive group. After the iminosugar moiety was bound to the target enzyme, the system was irradiated with UV light, resulting in covalent bonding of the probe to an active site amino acid residue. Probe **34** was successfully subjected to a photolabeling experiment against yeast α-gluocosidase and YgiK where the phosphine-FLAG ligand served as a reporting group, followed by SDS-PAGE and Western blot analysis. Furthermore, probes **35** and **36** displayed high specificity and sensitivity in the labeling of their corresponding target enzymes.

**Figure 13:**
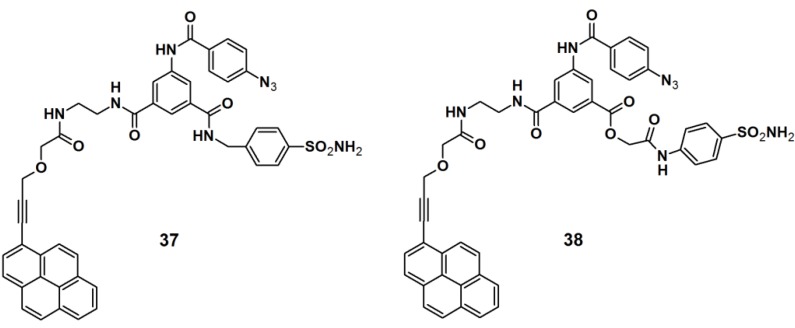
Arylazide-based photoaffinity probes **37** and **38** for HCA II.

PAPs for human carbonic anhydrase II (HCA II) were developed by Addy *et al.* [34]. The probes were designed as tripodal molecules by the use of 1,3,5-trisubstituted benzene as a scaffold (Figure 13). The synthesis commenced with the incorporation of a photoreactive arylazide unit to the 5-aminodimethyl isophthalate core via amide bond formation. Next, a propargylated pyrene derivative was coupled to the construct, which served as a reporting group. Additionally, a known inhibitor for HCA II, a sulfonamide derivative, was attached as a selective ligand. The labeling potential of probes **37** and **38** were investigated against HCAII inhibition by incubation with HCA II at various concentrations, followed by UV irradiation with a wavelength of 300 nm, directly followed by polyacrylamide gel electrophoresis (PAGE). The obtained results confirmed the successful labeling of both probes, as the expected fluorescent band was clearly present on the gel. The selectivity of the probes was determined by using a mixture of HCA II, bovine serum albumin (BSA), and the lysosome as a sample, where only the fluorescence band corresponding to HCA II was visible. Next, the probes were subjected to labeling experiments with *E. coli* cell lysates, where the selective capturing of HCA II was still clearly observed by SDS-PAGE analysis. 

**Figure 14:**
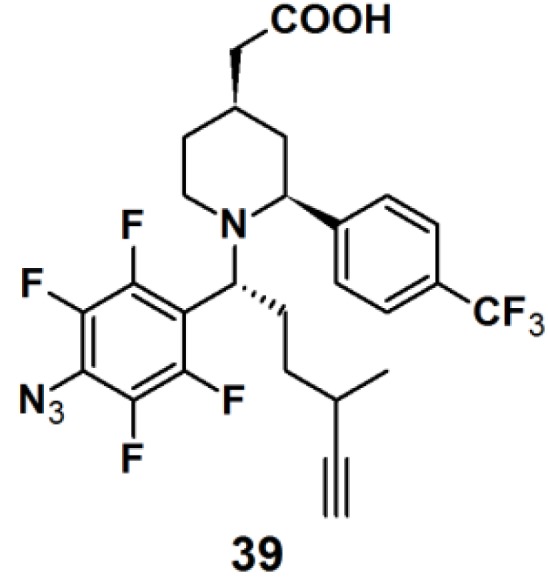
Arylazide-based photoaffinity probes **39**.

Probe **39** (Figure 14) was synthesized as a GSM-based PAP for detecting γ-secretase in the same series as described for probe **6** [22]. Both probes specifically and directly interacted with PS1ΔE9 (PS1 mutant), the catalytic subunit of γ-secretase, when subjected to experiments with PS1ΔE9 proteoliposomes using TAMRA-azide as a clickable reporting unit. When the PAL capability of PAPs **6** and **39** were tested with HeLa cells by employing biotin as a reporter tag, however, a more robust labeling was observed for probe **39** with respect to probe **6**. Probe **39** was subjected to another experiment with HeLa cells. In this case, however, TAMRA-azide was employed as the reporting group, which revealed a specific labeled band at 30 kDa, corresponding to PS1-NTF.

**Figure 15:**
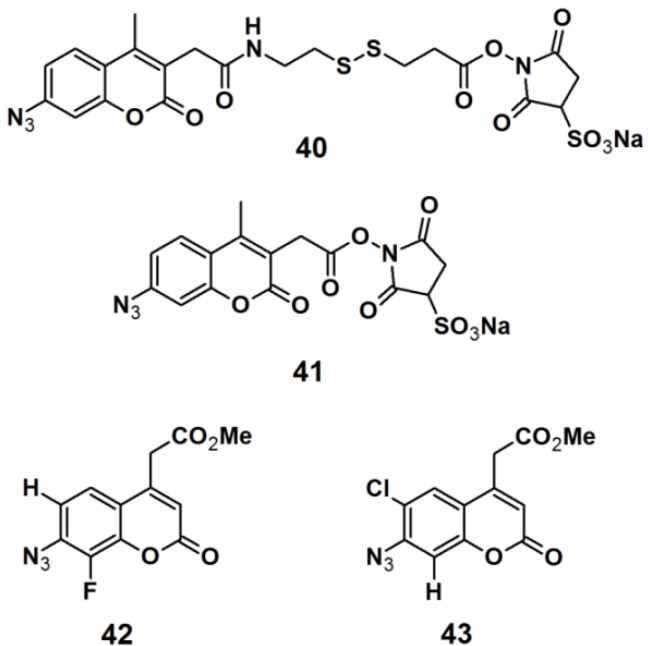
Arylazide-based photoaffinity probes **40**–**43**.

Pǎunescu *et al.* reported the development of small molecular size fluorophores based on a 7-azidocoumarin analog, as potential PAPs (Figure 15) [35]. The fluorophore structure was derived from the basic structure of the commercially available PAPs, sulfosuccinimidyl 2-(7-azido-4-methylcoumarin-3-acetamido)ethyl-1,3′-dithiopropionate (sulfo-SAED, probe **40**), and sulfosuccinimidyl-7-azido-4-methyl-coumarin-3-acetate (sulfo-SAMCA, probe **41**). These probes are currently used in protein modification, but suffer from low water-solubility and poor photostability. 7-Azidocoumarin is an interesting PAP, in the sense that it initially displays low fluorescence; however when the photo generated nitrene is trapped by insertion in either a C–H or N–H bond, the resulting 7-amino- or 7-hydrazinocoumarin is highly fluorescent. To overcome these drawbacks, the applied strategy was to incorporate a water-solubilizing agent, which is bioconjugatable in the 4-position in addition to a photoreactive azide functionality in the 7-position. Furthermore, the singlet nitrene intermediate which is generated upon UV-irradiation can be stabilized by decreasing the electron density of the coumarin ring; this can be accomplished by introducing an electron-withdrawing group to the coumarin core. In this report, electronegative halogen atoms such as a fluorine- and chloride atom were attached to either the 6- or 8- position of the coumarin derivative. The synthesized probes **42** and **43** were subjected to labeling studies in order to determine their photoreactivity upon irradiation with near UV light with a wavelength of 380 nm, in the presence of pyrrolidine as a nucleophile. Fortunately, both probes **42** and **43** underwent the desired N–H insertion reaction, thereby producing products with Stokes shift of 116 and 97 nm, respectively. This promising result renders the designed probes promising candidates for future designs of novel photoreactive cross-linkers intended for bio-labeling studies. 

**Figure 16:**
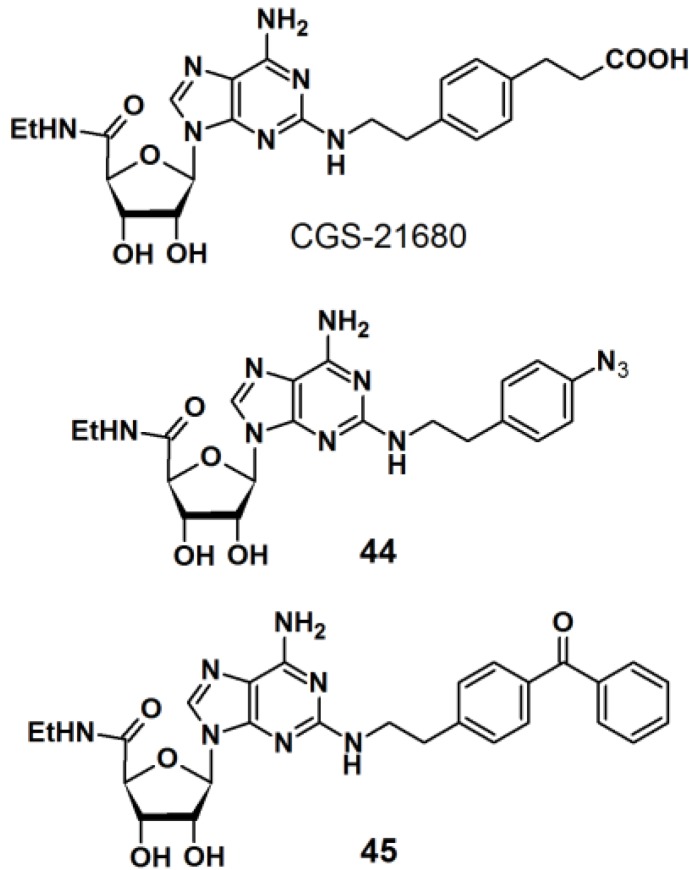
2-PEA based photoaffinity probes **44** and **45**.

PAPs based on 2-phenylethylamine have also been developed (Figure 16) [36]. 2-Phenylethylamine (2-PEA) is a basic component of many biologically active natural products. Analysis of the biological functions of 2-PEAs has been immensely pursued in the pharmaceutical field of research. 2-PEA has also been employed as a substructure of adenosine-receptor ligands. Adenosine receptors, consisting of four subtypes (A_1_, A_2A_, A_2B_, and A_3_), are G-protein-coupled receptors (GPCRs). In the brain, the A_2A_ receptors are highly expressed in the striatum. 3-{4-[2-({6-Amino-9-[(2R,3R,4S,5S)-5-(ethylcarbamoyl)-3,4-dihydroxyoxolan-2-yl]purin-2-yl}amino)ethyl]phenyl}-propanoic acid (CGS-21680) is a specific inverse agonist for A_2A_ receptors, resulting in hypotensive activity *in vivo*. An inverse agonist binds to the same receptor as an agonist, however, the activity of the receptor is decreased below its basal level by the inverse agonist. CGS-21680 has a 2-PEA substituent at the 2-position of adenine. The *p*-position of 2-PEA can be used for the introduction of functionalities on to the structure without drastically altering the pharmacological properties of the ligand. In the reported study, different photoreactive motifs such as phenyl azide, benzophenone, and (trifluoromethyl)phenyldiazirine derivatives were synthesized and incorporated into the *p*-position of the CGS-21680 core structure, instead of the usual carboxylethyl group. 2-PEA was subsequently functionalized with each of the aforementioned photoactive group at the *p*-position. The CGS-21680 core structure was designed as a 2-chloro-N-ethyladenosine-5′-uronamide derivative to enable the displacement of the 2-chloride atom with the photoreactive phenylethylamine derivatives. Unfortunately, the diazirine moiety did not survive the harsh coupling conditions and was omitted from this study, and only PAPs **44** and **45** were obtained. The synthesized photoreactive CGS-21680 derivatives were subjected to competitive binding assays with purified A_2A_R protein, in the presence of either an agonist or antagonist. Preliminary results indicated that both probes are active enough to justify further PAL studies of the A_2A_R protein. 

**Scheme 5:**
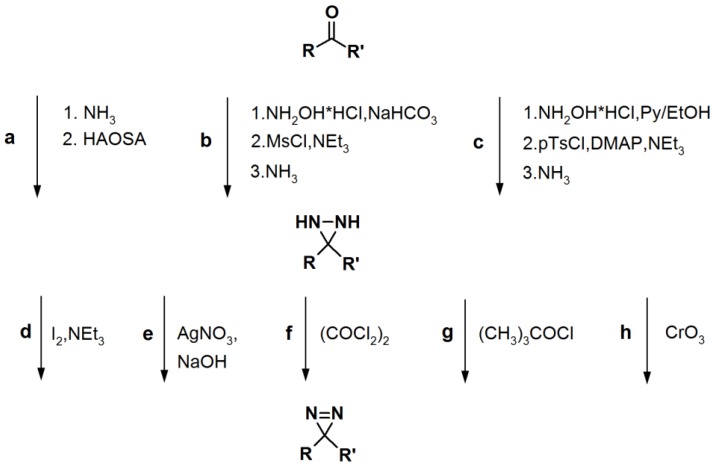
General synthetic route towards diazirine formation: HAOSA: hydroxylamine-O-sulfonic acid; MsCl: mesyl chloride; Py: pyridine; pTsCl: p-tosyl chloride; DMAP: *N*,*N*-dimethyl-4-aminopyridine [1].

### 2.3. Diazirine-Based Photoaffinity Labeling

Diazirine is the smallest molecular size photoaffinity group, which potentially results in low interference in the bioconjugation process. This characteristic makes it an attractive feature to incorporate their derivatives in the PAPs that target specialized proteins. Hence, diazirine derivatives have been widely explored as PAPs. The main strategy to synthesize diarizine analogs is the conversion of a derivatized ketone to the corresponding diaziridine, followed by the oxidation thereof to yield the diazirine analog. A summary of a typical diarizine synthesis is shown in Scheme 5.

**Figure 17:**
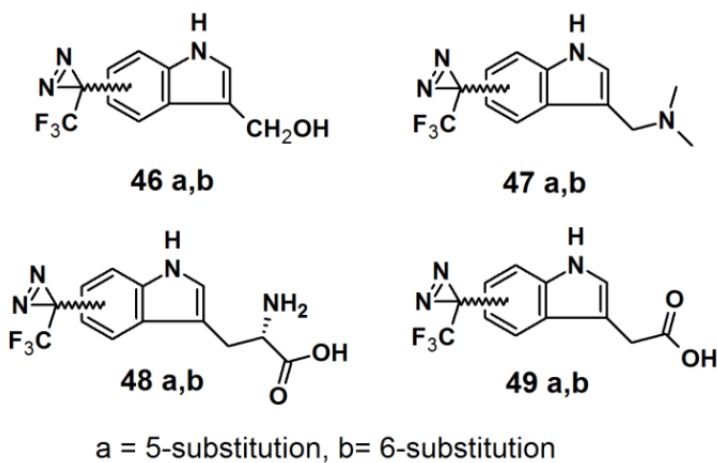
Diazirine-based photoaffinity probes **46**–**49**.

Murai *et al.* have developed PAPs based on the indole core structure (Figure 17) [37]. Indoles represent the basic component for a number of biologically active natural- and synthetic products; therefore this aromatic heterocycle has attracted enormous attention. 3-(Trifluoromethyl)-phenyldiazirine is reported to be relatively stable before irradiation and highly reactive after irradiation, yet results in few side reactions. As a consequence, 5- and 6-trifluoromethyldiazirinyl indole derivatives were synthesized in this study. The corresponding bromoindole was employed as a mother skeleton for the comprehensive synthesis of various bioactive indole metabolites. 

**Scheme 6:**
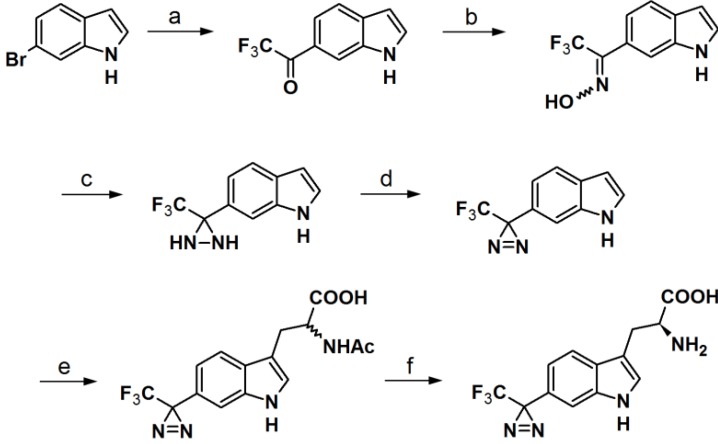
Synthesis of l-phototryptophan [37,41].

The synthesis entails the introduction of trifluoromethyldiazirizine at either the 5- or 6-position of the indole ring. Next, the indole core was further decorated with several side chains, at the 3-position. Probes **46a** and **46b** bear close resemblances to the indole carbinol structure, which is reported to possess anticarcinogenic, antioxidant and antiatherogenic effects. Probes **47a** and **47b** were designed to incorporate gramine derivatives, which typically plays a defensive role in plants. Probes **48a** and **48b** were developed to mimic diarizirinyl l-tryptophan. Normally, it is difficult to incorporate a diazirine functionality into the tryptophan structure due to the harsh conditions required for tryptophan synthesis [38,39,40]. This report describes more efficient and milder conditions, applicable in the synthesis of tryptophan (Scheme 6). Probes **49a** and **49b** contained indole-3-acetic acid (IAA), which is the main auxin in higher plant that has profound effects on plant growth and development. The synthesized probes were irradiated with a 100 W black-light in methanol to assess their photoaffnity response. All probes showed a decrease of the absorption intensity of near UV light with a wavelength of 380 nm, which indicated the complete disappearance of the diazirine rings. Probes **49a** and **49b** were also subjected to oat coleoptile segment growth bioassays. The results obtained by these studies indicate that the chemical modifications of IAA with a trifluoromethyldiazirinyl group at either the 5- or 6-positions are well tolerated by the target enzyme and do not hamper the biological activity of the newly synthesized probes.

*Reagents and Conditions*: (**a**) KH, THF, 0 °C/*t*-BuLi, CF_3_CO_2_Et, −78 °C; (**b**) NH_2_OH·HCl, Pyr/EtOH (7:3), 80 °C; (**c**) *p-*TsCl, Et_3_N, ether, 0 °C/NH_3_(*l*), ether, −78 °C; (**d**) PCC, pyr, DCM, rt or MnO_2_, ether, rt; (**e**) l-serine, Ac_2_O, AcOH, 75 °C or microwave, 45 °C, 40 W; (**f**) Aspergillus aminoacylase, phosphate buffer, 37 °C.

**Figure 18:**
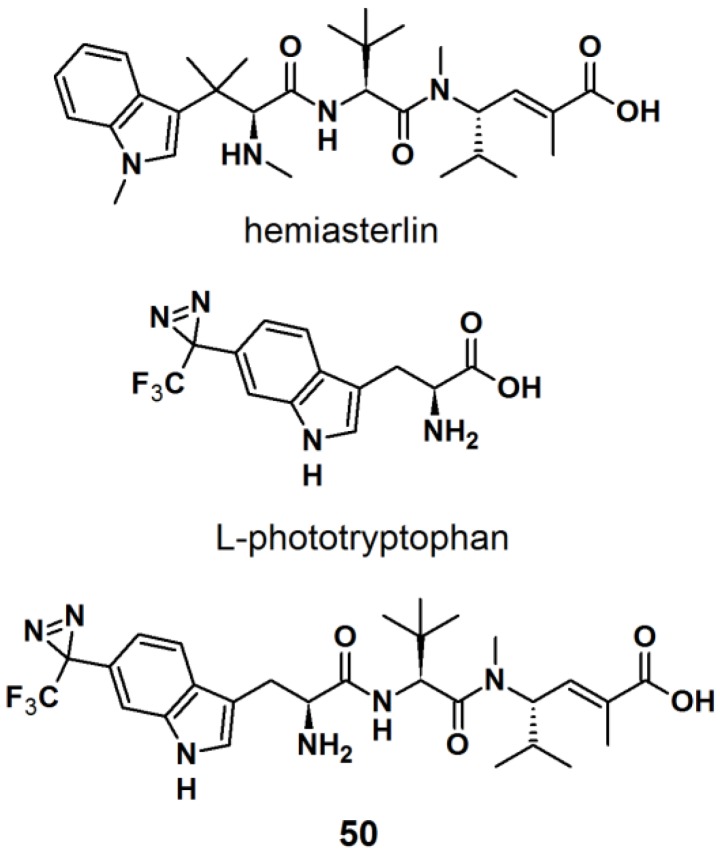
Natural product Hemiasterlin, l-phototryptophan and l-tryptophan PAP **50**.

The synthesis of an l-tryptophan-based PAP is also reported by Wartmann and Lindel [41]. These studies were mainly intended to incorporate different pharmacophores into the probes to avoid the alteration of their biological profile towards the target proteins. l-Tryptophan equipped with a diazirine unit at the 6-position was synthesized to give l-phototryptophan. The natural product hemiasterlin, originating from the marine sponges *Hemiasterella minor* and *Cymbastela* sp., containing an l-tryptophan motif was selected as a candidate structure for a PAP. The synthesis of l-phototryptophan was initiated with the trifluoroacetylation of 6-bromoindole at the 6-position. Next, the trifluoroacetylated indole was subjected to oxime formation with hydroxylamine hydrochloride in pyridine/ethanol. After tosylation of the obtained oxime derivative, the desired diaziridine analog was obtained in two steps involving reaction with liquid ammonia and subsequent oxidation. The trifluoromethyldiaziridine-derivatized indole was carefully reacted with l-serine to generate *rac*-N-acetylphototryptophan. The obtained racemic mixture was purified via enzymatic resolution with l-acylase to afford optically pure trifluoromethyldiazirinyl l–tryptophan. With l-phototryptophan in hands, probe **50** with a hemiasterlin-like structure was successfully synthesized by the coupling of a peptide segment to the l-phototryptophan modified diazirine probe under standard peptide coupling condition (Figure 18). The photoreactivity potential of the obtained probe was evaluated by irradiation with near UV light with a wavelength of 365 nm, which revealed the formation of an ether bond with methanol. 

**Figure 19:**
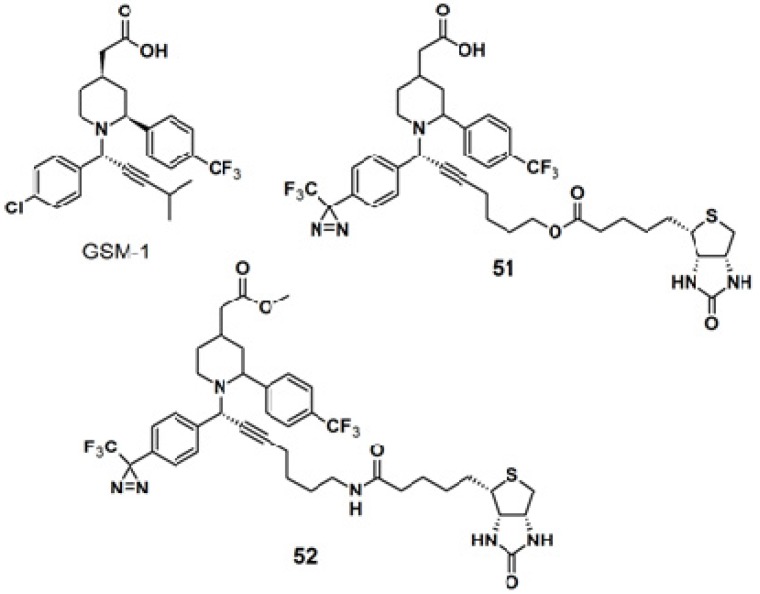
GSM-1 and diazirine-based photoaffinity probe **51** and **52**.

The diazirine functionality was also introduced to γ-secretase modulators, namely (GSMs)-based probes for targeting γ-secretase activity in Alzheimers disease-related research (Figure 19) [42]. The GSM-1 pharmacophore employed in this study belonged to the second generation GSMs, which are categorized into acidic- and non-acidic GSMs, by the presence or absence of a carboxylic acid. A metal catalyzed, ligand free, three-components coupling reaction (A^3^-coupling) [43,44,45] between a terminal alkyne, α-phenylamine, and benzaldehyde, in the presence of activated 4 Å-molecular sieves, was used to generate the GSM-1 derivative. After several optimization rounds, copper bromide was selected as the catalyst since it requires milder reaction condition in which the diazirine moiety remains intact. The straight probe **51** and the inverse probe **52** were both subjected to photolabeling experiments with N2a-ANPP cells and irradiated with near UV light with a wavelength of 350 nm followed by western blot analysis. Both probes were found to selectively label the N-terminal fragment of presenilin 1 (PS1), the catalytic subunit of the γ-secretase complex.

**Figure 20:**
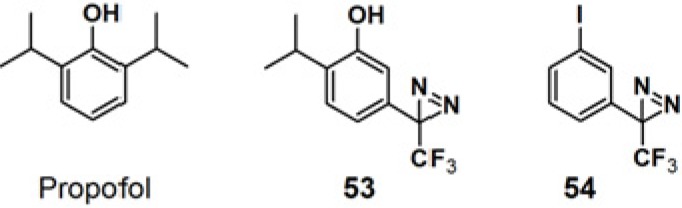
Propofol and diazirine-based photoaffinity probes **53** and **54**.

Propofol is a widely used intravenous general anesthetic, which acts at anesthetic concentrations as a positive allosteric modulator of γ-aminobutyric acid type A receptors (GABA_A_Rs), and at higher concentration as an inhibitor of nicotinic acetylcholine receptors (nAChRs) [46]. It is necessary to identify the propofol binding sites in GABA_A_Rs and nAChRs in order to determine whether propofol binds to equivalent or distinct binding sites in the Cys-loop receptors at different concentrations resulting in opposing effects, as a positive or negative allosteric modulator. PAP **53**, with a propofol analog, 2-isopropyl-5-[3-(trifluoromethyl)-3H-diazirin-3-yl]phenol (AziPm), was synthesized to characterize the propofol binding sites in a muscle-type *Torpedo* nAChR (Figure 20) [47]. 3-Trifluoromethyl-3-phenyldiazirine, which react with most amino acid side chains, including aliphatic side chains, was incorporated as a photoreactive group. The probe bears structural resemblance to PAP **54** (TID), a potent *Torpedo* nAChR inhibitor that binds to sites in the ion channel and in the δ subunit helix bundle [48,49,50]. The PAL experiment with probe **53** was executed by exposure to *Torpedo* nAChR-rich membrane, irradiated with near UV light with a wavelength of 360 nm and resolved after photolysis by Trisglycine SDS-PAGE and subsequently stained with Coomassie Blue R-250. Probe **53** was found to bind to three sites in the nAChR transmembrane domain: within the δ subunit helix bundle, in the ion channel, and at the γ-α interface. 

**Figure 21:**
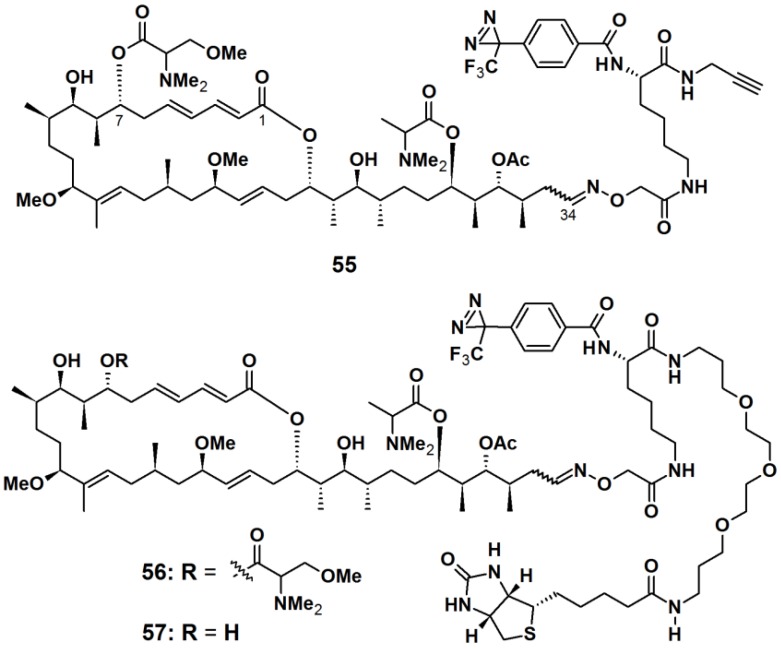
Diazirine-based photoaffinity probes **55**–**57**.

Aplyronine A (ApA), a 24-membered macrolide isolated from the sea hare *Aplysia kurodai*, exhibits *in vivo* antitumor activities against P388 murine leukemia cells and several cancer cells [51]. ApA targets actin by functioning as a depolymerizing agent and inhibits the polymerization reaction. Previous studies have established that the tail part (C24/C34) of ApA is important for its actin-binding property. Thus, the PAPs **55**, **56**, and **57** were designed to contain aplyronine A or C, where the photoreactive unit and the reporting unit or a terminal alkyne were incorporated at the tail region (Figure 21). ApA-based probes **55** and **56** were synthesized as a comparison between the ‘click on’ terminal alkyne and the attached PEG linked-biotin reporter, while probe **57** comprised of ApC and a PEG linked-biotin reporter. An aryltrifluoromethyldiazirine group was attached to the aplyronia unit via l-lysine (l-Lys) linkers. After incubation with actin and irradiation with near UV light with a wavelength of 365 nm, probe **55** was labeled with TAMRA-azide via an azide-alkyne Hüisgen cycloaddition reaction. The labeled actin was detected by fluorescence spectroscopy and Coomassie brilliant blue (CBB) staining, in which a highly fluorescent band corresponding to actin, was observed when the click reaction was applied for 30 min. Probe **56** was used to identify the target proteins of aplyronines in tumor cell lysate. Upon photoreaction followed by affinity purification using NeutrAvidin agarose, three strong bands, corresponding to β-actin and actin-related proteins (Arp2 and Arp3), were detected with silver stain as well as with anti-Arp2 and anti-Arp3 antibodies, respectively. These results demonstrated that there are specific interactions between the ApA analogs and actin or actin-related proteins. To confirm the binding properties between ApA and its target proteins, the resulting photo cross-linked products were subjected to streptavidin-HRP conjugate. A strong band corresponding to actin was observed upon treatment of the construct with probe **56**. In contrast, neither Arp2 nor Arp3 were detected as a biotinylated protein. Based on these results it was concluded that actin formed a covalent bond with probe **56**, while actin-related proteins did not. Furthermore, when probe **57** was subjected to affinity purification, both Arp2 and Arp3 were obtained with actin from cell lysate. It was concluded that actin-related proteins possibly interact with ApA via the actin/ApA complex.

### 2.4. Natural Photoaffinity Probes

**Figure 22:**
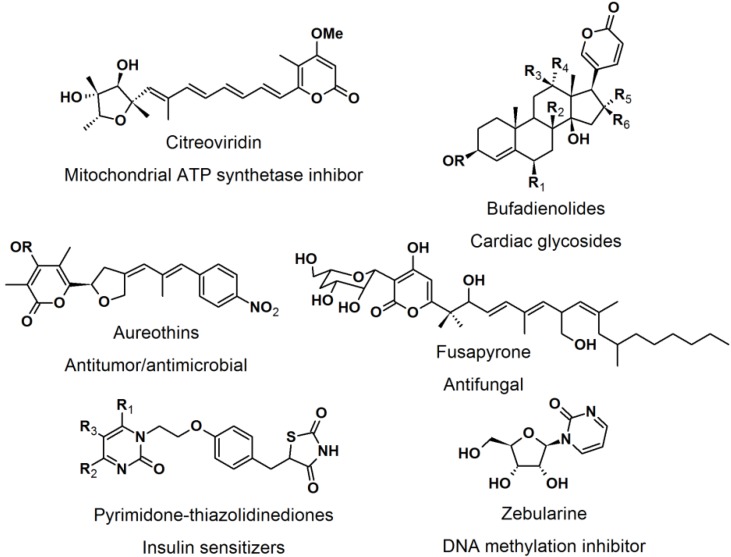
Pyrone and pyrimidone-containing bioactive compounds.

Other than the here described photoaffinity-probes, there have been reports on using a “more natural” type of photoreactive groups. [52] The purpose of the development of natural photoreactive probe or intrinsically photoreactive probes was to overcome the disadvantages of the synthetic photoreactive groups, which sometimes drastically perturb protein-ligand interaction. The natural photoactive probes contain photoreactive groups that generate highly reactive intermediates upon irradiation, for example steroid enones, diverse aryl chlorides, and several thioethers. Enones such as pyrones and pyrimidones are structural moieties found in many natural products and bioactive molecules, e.g., citreoviridin, bufadienolides, aureothins, fusapyrone, pyrimidine-thiazolidine diones, and zebularine (Figure 22).

**Scheme 7:**
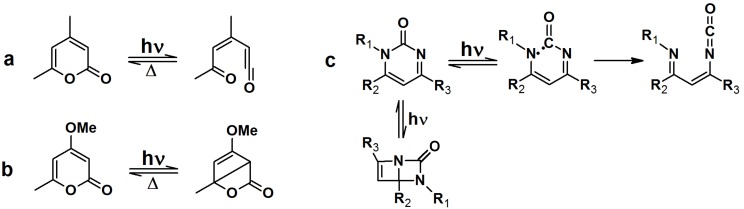
Light induced isomerization of pyrone and pyrimidone.

Upon irradiation with UV light, α-pyrones undergo an isomerization to either ketene or a bicyclic β-lactone, depending on the nature of the substituents on the pyrone ring (Scheme 7a,b). These isomers are susceptible to nucleophillic attack. Pyrimidone undergoes a Norrish type I reaction to form either an isocyanate or bicyclic intermediate which can react with nucleophiles (Scheme 7c). 

**Figure 23:**
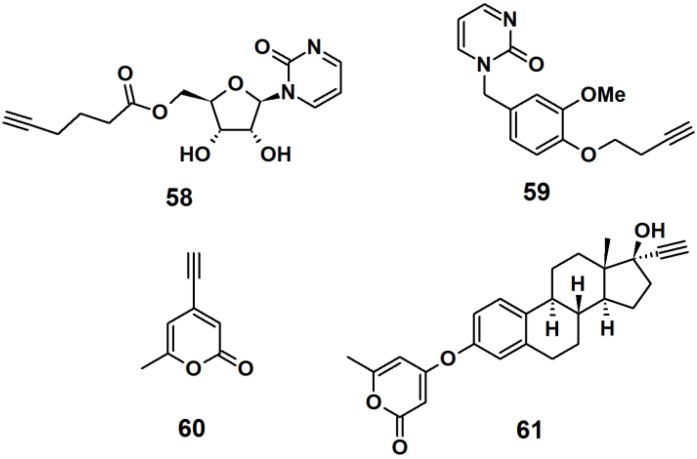
Examples of intrinsic photoaffinity probes.

Probe **58** was designed as a mimic of the DNA methylation inhibitor zebularine (Figure 23). The synthesis involved the formation of a β-*N*-glycosidic bond between a pyrimidine analog and peracetylated ribofuranose. Next, the acetyl groups were deprotected by ammonolysis in MeOH, and finally a terminal alkyne spacer was incorporated at the 5′-hydroxyl. Probe **59** was designed to resemble the dihydrofolate reductase inhibitor trimethoprim. The synthesis was achieved by attaching a terminal alkyne spacer to vanillin followed by the incorporation of the pyrimidine moiety to the vanillin scaffold to yield the desired probe. α-Pyrone probe **60** is reported to be a broad spectrum antibacterial agent and was prepared via bromination and subsequent Sonogashira cross-coupling reaction with the trimethylsilylacetylene group of the commercially available triacetic acid lactone. Probe **61** was based upon 17R-ethynylestradiol, a common estrogenic component in oral contraceptives that conveniently has a terminal alkyne in its structure. The core steroid structure possibly imparts specific protein binding in the bacterial proteome. 

**Figure 24:**
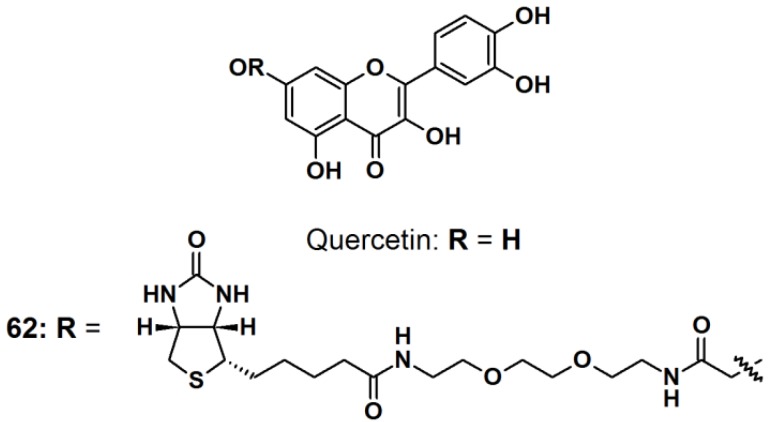
Quercetin intrinsic photoaffinity probe **62**.

Quercetin (Figure 24) is a natural product belonging to the flavonoid family, with multiple medicinal properties [53]. For instance, quercetin is reported to exhibit antioxidant-, anti-inflammatory-, and anticancer activity with almost no human toxicity. At the molecular level, quercetin has been found to inhibit many ATP binding enzymes and in particular, kinases, suggesting that part of its biological effects is due to the inhibition of signaling pathways, therefore quercetin has enjoyed extensive research in the pharmaceutical field. However, very little is known about the full range of proteins that quercetin targets and which targets are predominantly responsible for a particular biological effect. The reported study paid particular attention to quercetin’s well-known ability to inhibit the heat induction of heat shock protein 70 (HSP70). All quercetin derivatives used in reported study were previously synthesized and their capability of inhibiting casein kinase II (CK2) and Ca^2+^/calmodulin-dependent protein kinase II (CAMK2), which correlated to the ability to inhibit heat induction of HSP70, were characterized. Probe **62** was synthesized by incorporating PEG-linked biotin to the quercetin core structure by the use of an EDC/NHS-mediated coupling. The PAL experiments were carried out both *in vitro* and *in vivo*. In the *in vitro* study, an equimolar mixture of two readily available peptides, DRVYIHPFHL (angiotensin I) and RPKPQFFGLM (substance P), were irradiated with the probe. Liquid chromatography-coupled to tandem mass spectrophotometry (LC–MS/MS) analysis showed the formation of a cross-linkage between angiotensin I and a photo-fragment of the probe, providing proof of the successful photolabeling process. *In vivo* study was carried out by incubating probe **62** with Jurkat cells. The photo irradiation was done prior or after the cell lysis. When the cell lysates containing the probe were UV-irradiated for 30 min prior to pull down experiments and followed by the denaturing wash, discrete new bands were observed. Similar results were obtained when the cells were irradiated prior to lysis. Western blot analysis with a biotin-specific antibody was carried out to confirm that the proteins detected on the gel had been photo-modified. Only the samples that contained probe **62** and were irradiated showed bands with the anti-biotin antibody, which matched the major bands observed in the corresponding lane of the silver stained gel. Similar results were observed in the experiment in which the cells had been irradiated prior to lysis.

## 3. Conclusions

The field of photoaffinity labeling has been widely studied to identify the target(s) of biologically active molecules. PAPs based on photoreactive moieties such as benzophenones, aryl azides and diazirines, have been designed, synthesized and evaluated to assess their capability for being photoaffinity labels. Each type of photoreactive group has both advantages and disadvantages; hence the probes should be designed with consideration for both the target(s) and their ligand(s). Attempts to discover intrinsic PAPs have also been reported. Photoreactive components of natural molecules were utilized as probes to avoid complications associated with fully synthetic probes. Considering the wide varieties of target biomolecules and availability of a plethora of synthesis approaches towards PAPs, there is still room for researchers to further explore this research field.

## Selected Abbreviations and Acronyms

PAP: photoaffinity probe
PAL: photoaffinity labeling
SDS-PAGE: sodium dodecyl sulfate-polyacrylamide gel electrophoresis
TAMRA-azide: tetramethylrhodamine azide
APP: amyloid precursor proteins
GSMs: γ-secretase modulators
NSAIDs: nonsteroidal anti-inflammatory drugs
PTPs: protein tyrosine phosphatases
FITGE: two-dimensional gel electrophoresis
